# Specific absorption rate reduction for sub-6 frequency range using polarization dependent metamaterial with high effective medium ratio

**DOI:** 10.1038/s41598-022-05851-2

**Published:** 2022-02-02

**Authors:** Tayaallen Ramachandran, Mohammad Rashed Iqbal Faruque, Mohammad Tariqul Islam

**Affiliations:** 1 Space Science Center (ANGKASA), Universiti Kebangsaan Malaysia, UKM, 43600 Bangi, Selangor Malaysia; 2 Dept. of Electrical, Electronic & Systems Engineering, Universiti Kebangsaan Malaysia, UKM, 43600 Bangi, Selangor Malaysia

**Keywords:** Electrical and electronic engineering, Characterization and analytical techniques

## Abstract

This research study introduces a multi-layered square-shaped metamaterial (MSM) structure for the electromagnetic (EM) absorption reduction in wireless mobile devices. Usually, wireless devices, for example, a cellular phone emits radiofrequency (RF) energy to the surroundings when used it. Moreover, fast-growing wireless communication technologies that support cellular data networks have also motivated this study. Hence, the focus of the research was to reduce the Specific Absorption Rate (SAR) for the Sub-6 frequency range by designing a multi-layered and compact, 10 × 10mm^2^ sized metamaterial structure that can be attached inside a mobile phone by avowing any overlapping with existing parts. Overall, six distinct square-shaped metamaterials were constructed on 0.25 mm thick Rogers RO3006 substrate material to reach the target of this investigation. Furthermore, numerical simulations of the proposed metamaterial electromagnetic properties and SAR reduction values were performed by adopting Computer Simulation Technology (CST) Microwave Studio 2019 software. From these simulations, the proposed MSM structure exhibited multi-band resonance frequencies accurately at 1.200, 1.458, 1.560, 1.896 GHz (at L-band), 2.268, 2.683 2.940, 3.580 GHz (at S-band) and 5.872 GHz (at C-band). Simultaneously, the proposed MSM structure was simulated in High-Frequency Structure Simulator (HFSS) to authenticate the numerical simulation data. The comparison of simulation data shows that only the primary and last resonance frequencies were reduced by 0.02 and 0.012 GHz, whereas the rest of the frequencies were increased by 0.042, 0.030, 0.040, 0.032, 0.107, 0.080, and 0.020 GHz in sequential order. In addition, the introduced MSM structure manifests left-handed behaviour at all the resonance frequencies. Nevertheless, the highest recorded SAR values were 98.136% and 98.283% at 1.560 GHz for 1 g and 10 g of tissue volumes. In conclusion, the proposed MSM met the objectives of this research study and can be employed in EM absorption reduction applications.

## Introduction

Electromagnetic radiation is becoming a significant complication among wireless mobile phone users. As some of the radiation energy emitted by wireless devices is absorbed by tissues in the human body, thus it should be taken into serious consideration. Due to specific reasons, the concern about minimizing radiofrequency exposure in humans tends to be an uphill climb for mobile manufacturing companies. The measure of the energy absorbed rate by the human body is known as Specific Absorption Rate (SAR) and expressed in watt per kilogram (W/kg). According to the International Commission on Non-Ionizing Radiation Protection (ICNIRP) and the IEEE C95.1-2019 standards, the SAR restriction is limited to 2 W/kg averaged over 10 g (g) of tissue volumes^1,2^. A challenging problem that arises in this domain is how to decrease the SAR values at the desired resonance frequencies that comply with these guidelines. A material that has an extraordinary and superior accomplishment, referred to as a metamaterial, is responsible for the debate topic of conversation in the telecommunications field. This unconventional material is unable to find in a natural state and possesses unique effective medium parameters which are also difficult to gain in conventional materials^3^. Besides the SAR reduction application, metamaterial has also been utilised in many research fields, for instance, microwave application, satellite application, and sensor^4–7^. The stated latest research works have shown that, although the proposed scope of applications is varied for each other, the distinctive qualities of metamaterial allow the researchers to gain extraordinary outcomes. The studies on satellite application^4,5^ also become a hot topic in the scientific community. In these two research works, the author; Ramachandran et al. produce two distinct metamaterial structures on the same substrate material and size for C- and Ku band applications. Although the proposed metamaterial designs in these earlier research works possess great properties, obtaining a left-handed behaviour at all resonance bands are remains limited.

Numerous investigations have been performed to investigate the optimal electromagnetic absorption reduction values. Previous scientific works indicate that the unconventional material can control the SAR values that can be evaluated using respective computer software. In 2006, Hwang et al.^8^ introduced a square-shaped metamaterial design for electromagnetic reduction at 900 and 1800 MHz. The SAR reduction calculation was successfully performed in this study by adopting a finite-different time-domain method. A dipole antenna above the electromagnetic band-gap (EBG) substrate was suggested by Ikeuchi et al.^9^. Ikeuchi focuses on the SAR reduction at 3.50 GHz that covers the frequency bands until the 4G wireless communication system. Meanwhile, Kwak et al.^10^ introduced an optimised multilayer planar inverted-F antenna (PIFA) with the EBG structure to reduce undesired radiation that is exposed from the wireless device. Faruque et al.^11,12^ carried out two different investigations in this field in 2011 and 2014, respectively. The study^11^ focused on SAR computation in a muscle cube by utilising a square-shaped metamaterial with an adopted frequency range from 0 to 2.10 GHz. Meanwhile, Faruque^12^, designed a miniaturized double-negative triangular metamaterial to calculate the SAR values in the human body. Besides that, the scattering parameters were numerically simulated from the 0 to 1.20 GHz frequency range.

Even though there are numerous research studies in the SAR reduction field with different Global Systems for Mobile Communications, studies continue restricted in terms of performance. In 2014, Mat et al.^13^ investigate the SAR values that were manifested in the human head with ear implantation. Their study focused on three global system frequencies, namely 0.90 GHz, 1.80 GHz, and 2.10 GHz. The prosthesis was designed as a metallic bar and positioned homogeneously as a real ear attached to the human head. Alam et al.^14^ proposed a double-negative semi-circular radiating patch antenna with a 3 × 4 hexagonal-shaped metamaterial for mobile wireless applications. The numerically simulated SAR values were validated through a measurement process using the Satimo COMOSAR system. Meanwhile, Lee et al.^15^ proposed a SAR analysis of four different ages of anatomical head models and compared them with numerically simulated values obtained through the SAM phantom model adapted from SEMCAD X software. Concurrently, the SAR analysis was carried out for only two resonance frequencies of 0.835 GHz and 1.850 GHz while employing a typical bar-type cellular phone.

Previous research works can only be considered as a first step forward to a more profound understanding of how metamaterial affects the SAR reduction values from a different perspective. For example, Pandey and Singhal^16^ analysed the impacts of metamaterial structure with diverse sizes, distances, and locations on SAR values at 0.90 GHz resonance frequency in 2017. Pandey et al. proposed a circular-shaped split ring resonator on an FR-4 substrate material of 1.60 mm thickness. Metasurface unit cell for a polydimethylsiloxane-based flexible antenna was introduced by Janapala et al.^17^. Two different leaf structured antennas with design, i.e., without and with metasurface were analysed in the study at the 2.40 GHz WLAN ISM band. Meanwhile, Ramachandran et al.^18^ investigate the SAR reduction values for just two resonance frequencies that fell in the L- and S-bands, respectively. A substrate material with a dimension of 11 × 11mm^2^ and 1.60 mm thickness was utilised in the study which employed a frequency range from 0.30 to 4 GHz. Besides that, Chaudhary and Vijay^19^ studied the effect of the antenna on SAR values when placed near the human head. For the electromagnetic absorption reduction application, the authors successfully introduced a shielding material to absorb radiation from the wireless device. An analysis of diverse positions of antenna integrated into the wireless device and various material shields was conducted in their study. Meanwhile, another interesting topic of metamaterial absorbers is also widely investigated by many researchers around the world. This type of metamaterial can manifest multiple resonance frequencies and be able to integrate with terahertz frequencies^20–23^.

The multi-layered metamaterial also becoming a hot topic among scientists (e.g.^24,25^). Commonly, this type of metamaterial is composed of multi altering layers of substrate materials that are used in a wide range of applications such as sensors, absorption, perfect imaging, coatings for tailored emittance, etc. However, in this research work, the authors introduced a multi-layered metamaterial structure with similar altering substrate layers to overcome cost constraints for the EM absorption reduction application. The preliminary outcome of this research work indicates that the multi-layered metamaterial structure has a great influence on the SAR reduction values. Although previous studies regarding this issue possess substantial contributions in decreasing the SAR values discharged from mobile phones, the rising latest technologies require a progressive method or structure to better minimize the radiation effect. Thus, in our review, the previously reported literature works suffer from certain weaknesses. For instance, the recently introduced 5th generation wireless communication technology requires an improvement in mobile phones to minimize the radiation effects. The 5G network is divided into two categories, particularly, frequency range 1 (FR1) and frequency range 2 (FR2). The FR1 applied for sub-6 bands whereas, FR2 was used for communication at the millimetre-wave frequencies above 24 GHz. The safety guidelines and standards set by the ICNIRP, and IEEE will assure the mobile phones never exceed the highest allowable exposure level when working in the utmost practicable circumstance. The ICNIRP 2020 guidelines have mostly retained the similar restrictions that are set in 1998. Nevertheless, the ICNIRP 1998 guidelines have major restrictions that are strongly inappropriate for the latest technology evolution, for example, the 5G mobile network. A similar six-minute averaged SAR basic restriction was used in both guidelines to ensure the protection against excessive local temperature rise. Although, the SAR averaged over a 10 g contiguous tissue region is essential in ICNIRP 1998, while the latest guidelines require it to be averaged over a 10 g cubic region. This change is necessary to influence the better approximation of temperature rise. The 10 g cubic mass is used because heat diffusion evenly spreads the thermal energy to a much larger volume. Meanwhile, for local exposure, the time interval is implemented as a six-minute average since it corresponds with the thermal time constant. Furthermore, for the frequency below 6 GHz, the guidelines present distinct exposure limits for each body part. Therefore, the focus is to maintain the SAR restriction by constructing a metamaterial shield that can be integrated inside a wireless device. The shielding here defines as a barrier to prevent the electromagnetic energy emitted from the mobile phone from penetrating the human head. The physical and effective medium properties of the proposed MSM design are the main factors to utilise in a wide range of applications. A wireless device with a metamaterial structure will be examined while operating at its maximum power level in all resonance frequencies to find the optimised results. This investigation aims to construct an unconventional material with nonuple-resonance frequencies that possess left-handed behaviour.

## Materials and simulation methods

### Multi-layered metamaterial design construction

**Figure 1:**
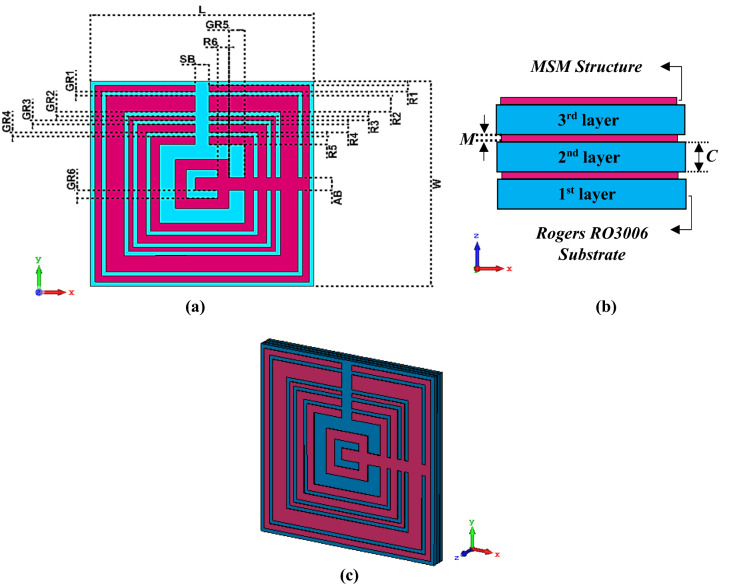
The MSM structure: **(a)** top view, **(b)** front view, **(c)** isometric projection view.

In the computer-based simulation process, the construction of the MSM design (as shown in Fig. 1a–c) acts as a crucial function in gaining the required electromagnetic properties. The proposed MSM unit cell design was initially made up of Rogers RO3006 substrate material of length, L = 10 mm and width, W = 10 mm. This dielectric substrate material with thickness, C = 0.25 mm as illustrated in Fig. 1b was selected in this research study. In the selection process of substrate material, the tangent loss (δ) and dielectric constant (ε) of the RO3006 material were taken into the consideration. This type of substrate material has a low dielectric loss and can describe as excellent mechanical properties versus temperature. Therefore, the Rogers RO3006 is ideal for reliable stripline and multi-layer board design construction with a range of dielectric constant. Typically, this substrate material is applied in a wide range of applications, such as cellular telecommunications systems, global positioning satellite antennas, direct broadcast satellites, remote meter readers, automotive radar applications, etc. This research is completely based on novel six square metamaterial rings with distinct gaps and dimensions that were built on the Rogers RO3006. For the construction of MSM structure, annealed copper material with conductivity, σ = 5.80 × 10^7^ S/m was selected, and the thickness was decided to fix as 0.035 mm from the surface of RO3006 material. The first or the largest square ring was constructed with a width of 0.30 mm. Meanwhile, the first five square rings have similar gaps of 0.20 mm between the rings. The second to fifth square rings have widths of 0.80, 0.20, 0.40, and 0.40 mm, respectively. A rectangular bar with length and width of 3.50 mm and 0.60 mm, respectively, was subtracted from the top of the square ring structure. Therefore, with a gap of 0.70 mm, the last ring was built with a width of 0.50 mm. A rectangular bar with a length and width of 5 mm and 0.60 mm was added after a gap of 0.40 mm from the last ring. Lastly, a similar unit cell metamaterial design was placed twice at the back substrate material to form a multi-layered structure. These square-shaped rings were built accordingly to obtain multi resonance frequencies. Meanwhile, the MSM isometric projection view is demonstrated in Fig. 1c. Table 1 describes the dimension lists of the MSM unit cell metamaterial design.

**Table 1:** Description of proposed MSM unit cell metamaterial.

Descriptions	Dimension (mm)
First ring, R1	0.30
Second ring, R2	0.80
Third ring, R3	0.20
Fourth ring, R4	0.40
Fifth ring, R5	0.40
Sixth ring, R6	0.50
GR1–GR4	0.20
GR5	0.70
GR6	0.40
Additional bar width, AB	0.60
Subtracted bar width, SB	0.60
Substrate length, L	10.00
Substrate width, W	10.00
Metamaterial thickness, M	0.035
Substrate thickness, C	0.250

*GR* gap between rings.

### Computer simulation technique

**Figure 2:**
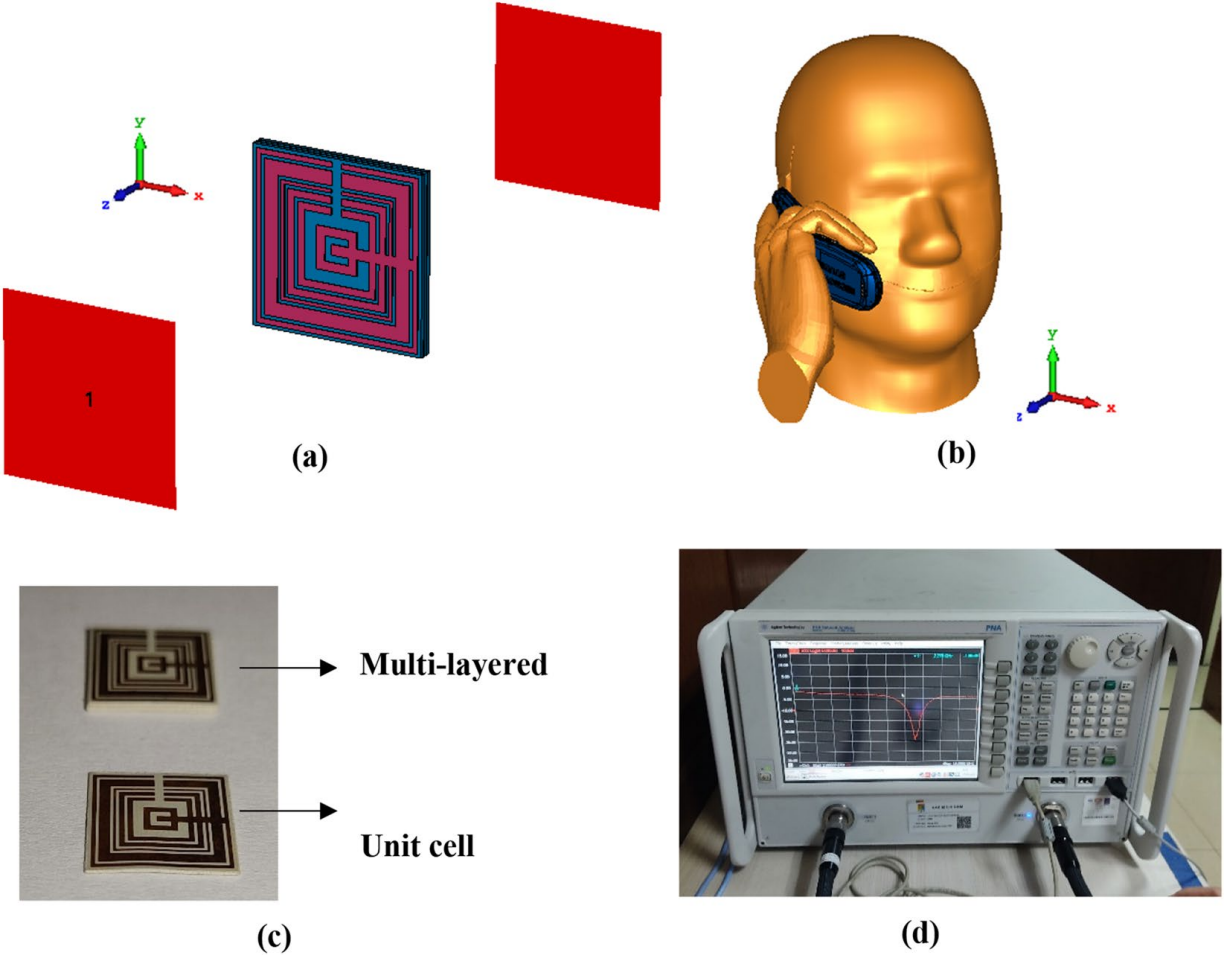
Numerical simulation geometry: **(a)** MSM structure, **(b)** Voxel human head and hand model, **(c)** Fabricated MSM structure, **(d)** Agilent N5227A VNA instrument.

The simulation of the proposed metamaterial was carried out by adopting commercially available software known as Computer Simulation Technology (CST) Microwave Studio 2019^26^. Generally, CST software is utilised in electromagnetic problem solving with accurate outcomes. The numerical simulations were comprised of three stages, namely MSM simulation, SAR reduction (in CST), and MSM simulation (in High-Frequency Structure Simulator 15.0 (HFSS)^27^). The first stage was numerically carried out by using a frequency-domain solver and tetrahedral mesh. The MSM structure was placed at the middle of waveguide ports as displayed in Fig. 2a. Moreover, the ports were located at the positive and negative z-axis by adopting Transverse Electromagnetic wave (TEM). Subsequently, the Perfect Electric Conductor (PEC) boundary condition was set for the x-axis, while the y-axis was set as Perfect Magnetic Conductor (PMC). Because this research aims to cover the latest wireless communication technology, the frequency range was set from 0 to 6 GHz. Although 5G is on its way, many countries are still using the 4G network. Therefore, this primary interest led to the examination of electromagnetic absorption reduction values at the selected frequency range. The focus of this simulation was to calculate the scattering parameters of the proposed MSM design. The obtained reflection (S11) and transmission (S21) coefficient results were employed to compute the effective medium parameters of the MSM structure, i.e., permeability (μ), permittivity (ε), refractive index (n), and impedance (z) values. The famous Robust method was adopted for this calculation to find the superior characteristics of the metamaterial by utilising MATLAB 2021 software^28–30^. The retrieval equations of impedance (z), refractive index (n), permittivity (ε), and permeability (μ) using the Robust method are defined in Eqs. (1) to (4). The proposed metamaterial structure needs to characterize as an effective medium homogeneous to retrieve the effective permittivity and permeability values. Furthermore, the permittivity and permeability values were retrieved from the S11 and S21 data. The *d* in Eq. (2) refers to the thickness of the metamaterial slab. Meanwhile, *d* of 1.635 mm is utilised in this retrieval process. Lastly, the S21 results were validated in HFSS software by utilising the same methods and techniques as in CST.

1 $$z=\pm \surd \frac{{(1+{S}_{11})}^{2}-{S}_{21}^{2}}{{(1-{S}_{11})}^{2}-{S}_{21}^{2}}$$

2 $$\begin{gathered} n = \frac{1}{{k_{0} d}}\left\{ {\left[ {In\left( {e^{{ink_{0} d}} } \right)} \right]^{{\prime \prime }} + 2m\pi - i\left[ {In\left( {e^{{ink_{0} d}} } \right)} \right]^{{\prime \prime }} } \right\} \hfill \\ e^{{ink_{0} d}} = \frac{{S_{{21}} }}{{1 - S_{{11}} \frac{{z - 1}}{{z + 1}}}} \hfill \\ \end{gathered}$$

3 $$\varepsilon =\frac{n}{z}$$

4 $$\mu =nz$$

The second stage was the numerical analysis of electromagnetic absorption reduction to identify the effect of the metamaterial attachment inside the mobile phone. Specific Anthropomorphic Mannequin (SAM) Phantom available in CST Microwave Studio was adopted for this objective. The simplified SAM phantom that is homogeneous to the real human head model based on the IEEE standard is composed of two components, namely an outer shell and fill with a tissue-stimulating liquid substance. Furthermore, the SAM phantom represents 90% of the male population’s head size for a standardised certification objective. A simple flip mobile phone was adapted and positioned at the right-hand side of the human head with a hand model to hold it in talk mode (as shown in Fig. 2b). The adapted mobile phone was embedded with a keyboard, antenna, circuit, LCD components, and housing. The dielectric properties of the head model, tissue-stimulating liquid at three resonance frequencies, and the phone model are presented in Table 2.

**Table 2:** Dielectric properties of the phone material and SAM phantom.

	ε_r_	σ(S/m)
**Phone parts and materials**		
LCD glass	4.78	–
LCD cover pad	3.50	0.005
Circuit PCB	4.90	–
Housing	2.50	0.005
Keyboard	3.50	0.005
**SAM phantom**		
Headshell	3.70	0.0016
Liquid 0.915–1.450 GHz	41.47	0.98
Liquid 1.450–1.610 GHz	40.50	1.20
Liquid 1.800–1.950 GHz	40.00	1.40
Liquid 2.100–2.450 GHz	39.82	1.49
Liquid 2.450–3.000 GHz	39.20	1.80
Liquid 3.000–5.000 GHz	38.72	2.43
Liquid 5.200–6.000 GHz	36.00	4.70

The SAR exposure value of the mobile phone with and without the MSM attachment was calculated using a time-domain solver. The distinct position of the metamaterial structure inside the wireless device was chosen through a parametric analysis (further details in the array metamaterial position in mobile phone subsection). The centre point of the antenna structure in the mobile phone was aligned along the central right cheek area. When a mobile phone is held in a talking mode by the user, then the head surface is often not in a flat position. Therefore, the distance between the antenna and the human head model was roughly 20 mm. Furthermore, the default 0.50 W peak reference power was applied in this simulation process, enabling the power to surge through into the MSM design. Eventually, at the feeding spot, the reflection is lost, thus the calculation of the absorption reduction values is not influenced by the default power. Fundamentally, in the deficiency of the ohmic loss condition, the antenna radiated power is corresponds to the reference power. Therefore, the calculation of SAR value for averaged over 1 g and 10 g of tissue volumes, the power-loss density was selected. Meanwhile, in the last stage, the proposed metamaterial was simulated using HFSS software to validate the numerical simulation results. A similar simulation method as in the CST software was adopted for this simulation process. On the other hand, the proposed unit cell and MSM metamaterial designs were fabricated as illustrated in Fig. 2c. For the validation purpose, the fabricated unit cell metamaterial was undergone a measurement process by utilising Agilent N5227A Vector Network Analyser (VNA) as demonstrated in Fig. 2d. Before measurement was carried, the VNA instrument was calibrated by using the Agilent N4694-60001 device.

## Results and discussions

**Figure 3:**
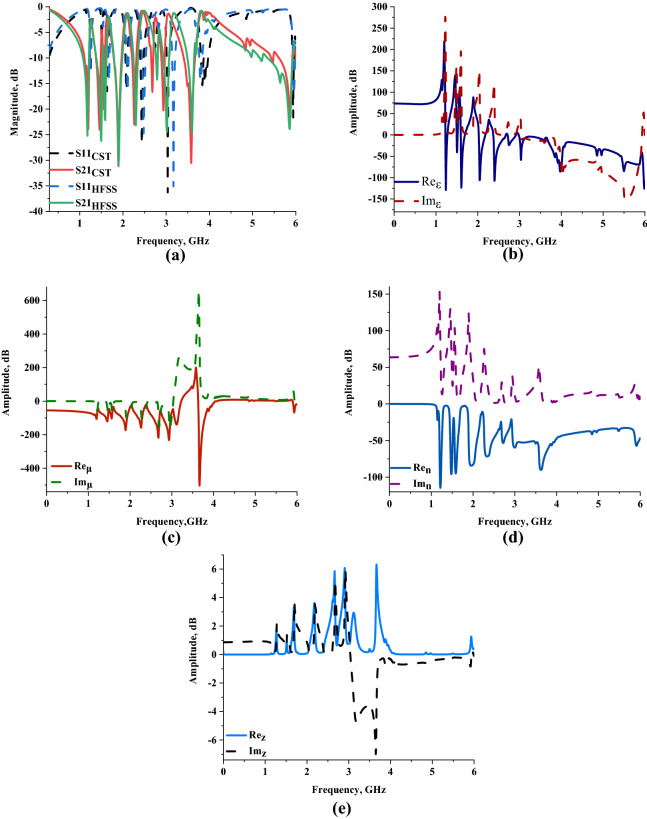
Scattering (from CST & HFSS) and effective medium parameters of the MSM design (from MATLAB): **(a)** Numerically simulated S11 and S21 results, **(b)** Permittivity, **(c)** Permeability, **(d)** Refractive index, **(e)** Impedance value.

Figure 3a–e illustrate the plots of results that were obtained from the computer simulation such as S11, S21, permittivity (ε), permeability (μ), and refractive index (n). Our findings in Fig. 3a demonstrates the outcome of numerical simulation on z-axis wave propagation with multi-resonance frequencies in L-, S- and C-bands precisely at 1.200, 1.458, 1.560, 1.896, 2.268, 2.683, 2.940, 3.580 and 5.872 GHz with tolerable magnitude values of − 23.862, − 23.991, − 20.068, − 31.109, − 23.026, − 16.526, − 20.204, − 30.363, and − 21.788 dB, respectively. We also describe the reflection and transmission coefficient outcomes obtained from the HFSS software in Fig. 3a. The comparison showed moderately dissimilar resonance frequencies of 1.180, 1.500, 1.590, 1.900, 2.300, 2.790, 3.020, 3.600, and 5.860 GHz, respectively. Meanwhile, this numerical simulation data reveals acceptable magnitude values such as − 25.208, − 26.243, − 22.194, − 30.951, − 23.203, − 14.263, − 24.422, − 24.636, and − 23.916 dB. In summary, there was a mean difference of 1.729% between both numerical methods for all resonance frequencies. However, the numerically simulated S11 and S21 results also demonstrate contrasting outcomes. This is because all power is not transmitted or reflected throughout the simulation. A vital discovery arises that the permittivity values of both substrate material and copper structures contribute significant advantages to gain distinct results. Each resonance frequency that the metamaterial structure obtained was controlled by these permittivity values. Besides that, when the dielectric constant values increase, it persuaded the capacitance value at the radiating element interval and the ground.

Our findings on the permittivity values illustrated in Fig. 3b clearly show eight negative permittivity characteristics at all resonance bands. At the L-band, the frequency ranges from 1.230 to 1.270 GHz, 1.500 to 1.518 GHz, and 1.596 to 1.692 GHz have the negative permittivity behaviour with tolerable below-zero amplitude values of − 63.019 to − 4.294 dB, − 30.326 to − 4.104 dB, and − 7.946 to − 0.756 dB, respectively. Meanwhile, another four negative permittivity values also occurred at the S-band. For the frequency range from 2.028 to 2.178 GHz and 2.376 to 2.670 GHz, the proposed MSM design exhibits a negative characteristic with amplitude values from − 5.434 to − 1.828 dB and − 3.081 to − 0.766 dB, respectively. Furthermore, ranges from 2.718 to 2.916 GHz and 2.988 to 3.996 GHz, the MSM design manifested negative permittivity values with maximum amplitude values of − 23.957 dB and − 86.048 dB, accordingly. The last resonance frequency band, the proposed MSM design exhibits negative behaviour at the frequency range from 4.002 to 6.000 GHz with acceptable magnitude values from − 78.646 to − 108.962. These results highlight that the maximum permittivity value took place at the frequency of 1.236 GHz with an amplitude value of − 129.104 dB. Additionally, the MSM metamaterial has five negative permeability characteristics at all resonance bands as demonstrated in Fig. 3c. Essentially, with a range difference of 0.996 GHz, starting from 1.002 GHz has amplitude values ranging from − 69.246 to − 37.961 dB. On the other hand, the following bands have two negative characteristics for each, respectively. In the S-band, negative permeability values were observed from 2.004 to 3.222 GHz and 3.636 to 3.996 GHz with the amplitude values moderately changed between − 34.455 to − 4.384 dB and − 20.900 to − 14.318 dB. Finally, in C-band, the proposed metamaterial manifest two small frequency ranges from 4.002 to 4.080 GHz and 5.898 to 6.000 GHz with acceptable amplitude values.

A further optimistic finding from these results is that the proposed MSM design possesses a left-handed behaviour for all three frequency bands. The fundamental theory of the left-handed behaviour defined, besides the refractive index possesses negative amplitude values, the permittivity and permeability values as well have the same performance. For illustrative purposes, the frequency range of the initial negative permittivity claimed before has left-handed behaviour with permeability and refractive index (like demonstrated in Fig. 3d) ranges from − 39.236 to − 63.825 dB and − 105.638 to − 23.109 dB, respectively. All the frequency ranges of the left-handed characteristic are tabulated in Table 3. The S-band has the highest number of frequency ranges that possess left-handed behaviour.

**Table 3:** Frequency ranges of left-handed characteristics for all resonance bands.

No	Frequency range (GHz)	Permittivity, dB	Permeability, dB	Refractive index, dB
1	1.230 to 1.272	− 63.018 to − 4.294	− 39.236 to − 63.825	− 105.638 to − 23.109
2	1.500 to 1.518	− 30.323 to − 4.104	− 46.981 to − 64.195	− 80.399 to − 38.026
3	1.596 to 1.692	− 7.936 to − 0.756	− 34.477 to − 73.707	− 93.093 to − 13.102
4	2.028 to 2.178	− 5.434 to − 1.828	− 24.284 to − 93.539	− 79.672 to − 17.850
5	2.376 to 2.670	− 3.081 to − 0.766	− 23.100 to − 201.480	− 70.547 to − 24.064
6	2.718 to 2.916	− 1.294 to − 0.003	− 61.983 to − 191.707	− 53.395 to − 20.875
7	2.988 to 3.222	− 2.419 to − 4.034	− 76.954 to − 53.751	− 59.348 to − 4.384
8	3.636 to 3.996	− 6.143 to − 76.779	− 20.900 to − 14.318	− 89.853 to − 47.999
9	4.002 to 4.080	− 78.646 to − 27.381	− 12.748 to − 0.636	− 47.565 to − 44.705
10	5.898 to 6.000	− 52.295 to − 108.962	− 3.174 to − 19.400	− 55.745 to − 46.900

Generally, the left-handed inspired metamaterial can be implemented in a wide range of fields, such as SAR reduction, radome, antenna, satellite application, coupler, etc. The unique left-handed characteristic usually maximises the accomplishment of the material, for example, propagation in the backward wave, negative refraction index, and reversed Doppler shifts. Furthermore, Fig. 3e indicates the impedance values computed using CST software. Nine maximum impedance values were manifested in this research work at all resonance bands. The impedance value achieved a peak value in the L-band, for instance, 1.863, 0.720, 3.361Ω at 1.272,1.524, and 1.692 GHz, respectively. Meanwhile, the maximum point reached 3.631, 5.865, 6.087, 2.943, and 6.323Ω at 2.178, 2.664, 2.904, 3.120, and 3.666 GHz for the S-band frequency range. The last impedance peak value was occurred at near to 6 GHz with 1.272 Ω. It is clearly shown that the impedance frequencies were increased when compared to the resonance frequencies. Therefore, the impedance has three peak frequencies while the metamaterial manifest four resonance in L-band. Meanwhile, in S-band, the impedance possesses five peak frequencies while the metamaterial is just four only. As the imaginary refractive index and real impedance values exhibited more than zero, the MSM structure is possibly categorised as a passive medium design. The effective medium parameter is an analysis calculation to determine the impact on the metamaterial during the external time-variant electromagnetic fields exposure.

Commonly, the relationship between magnetic and electric responses is related to the physical characteristic of the electromagnetic field. This is because the incident happens in space due to the electric charges which have a time-varying response. The static charges are only generated in space with the static fields. Meanwhile, the source of time-varying electric charges provides a high chance for magnetic field improvement and eventually generates time-variant electric fields. Usually, most materials fall under two classifications, for instance, dispersive and lossy materials. In general, these materials exhibit superior permittivity, permeability values and as well finest resonance frequencies.

**Figure 4:**
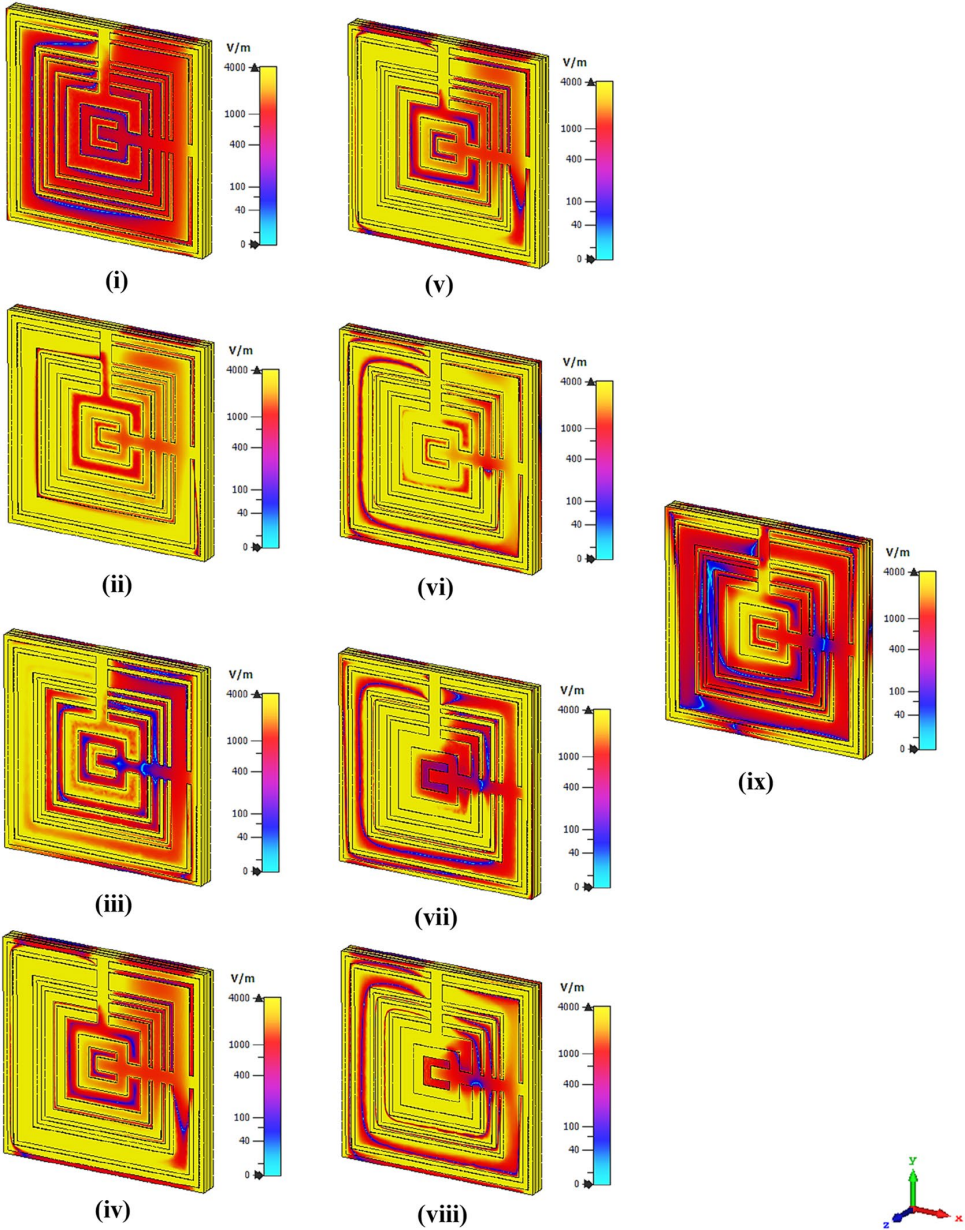
Absolute electric field distribution of the proposed MSM design at; **(i)** 1.2 GHz, **(ii)** 1.458 GHz, **(iii)** 1.56 GHz, **(iv)** 1.896 GHz, **(v)** 2.268 GHz, **(vi)** 2.683 GHz, **(vii)** 2.940 GHz, **(viii)** 3.580 GHz, **(ix)** 5.872 GHz.

**Figure 5:**
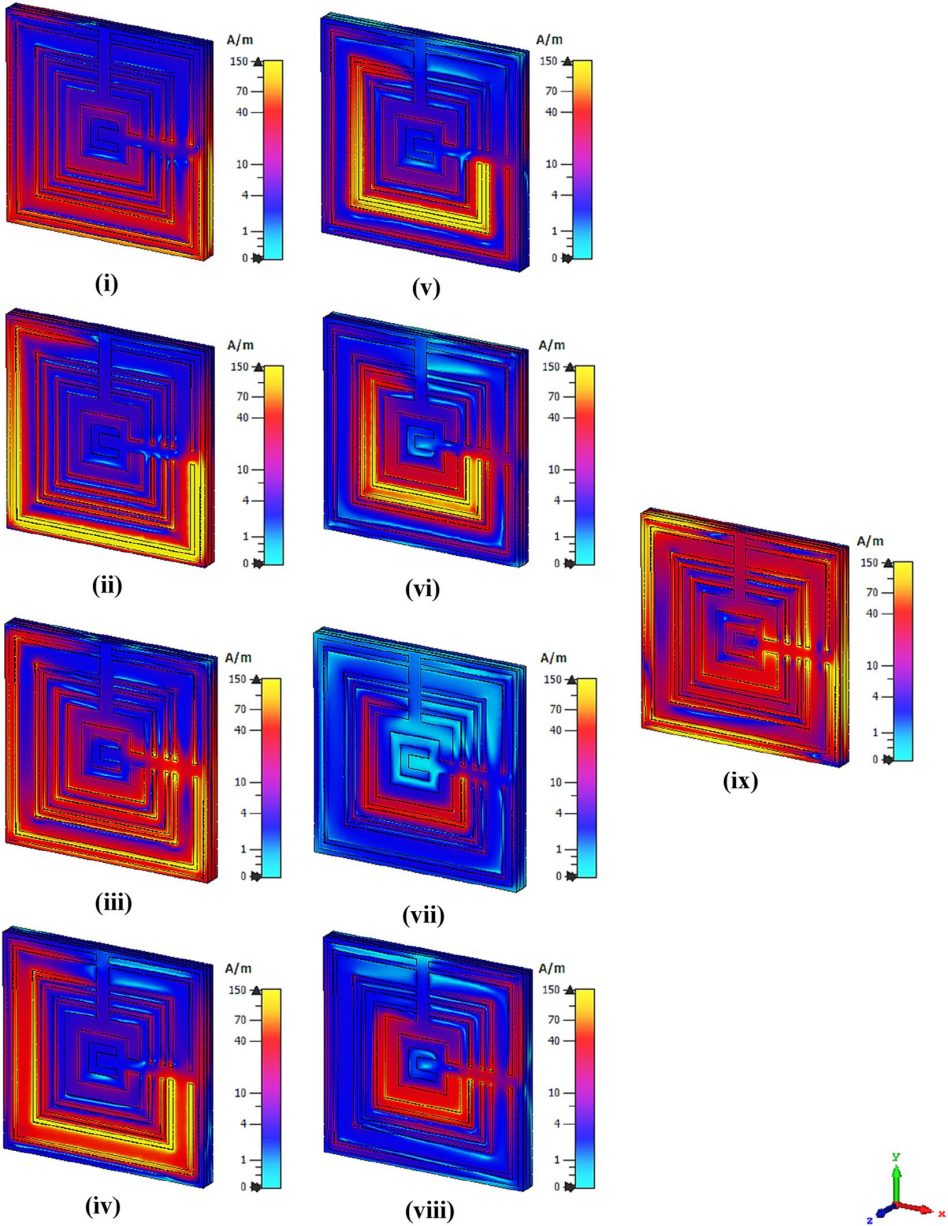
Absolute magnetic field distribution of the proposed MSM design at; **(i)** 1.2 GHz, **(ii)** 1.458 GHz, **(iii)** 1.56 GHz, **(iv)** 1.896 GHz, **(v)** 2.268 GHz, **(vi)** 2.683 GHz, **(vii)** 2.940 GHz, **(viii)** 3.580 GHz, **(ix)** 5.872 GHz.

The absolute electric and magnetic field distributions created on the MSM metamaterial structure at nine resonance frequencies are displayed in Figs. 4 and 5, respectively. The initial review displays that a powerful electric field distribution (EFD) that happened for nine resonance frequencies was displayed nearly the whole MSM design. All the resonance frequencies except 1.200 and 5.872 GHz have EFD that was concentrated all over the structure as shown in Fig. 4. Meanwhile, the MSM metamaterial possesses far fewer magnetic field distribution (MFD) compared to EFD. During the occurrence of the magnetic response, the magnetic field induced current in the rings when the wave propagates through the split ring resonator structure. The 5.872 GHz resonance frequency has a higher MFD compared to the other resonance frequencies. For the rest of the resonance frequencies, the signs of MFD were indicated on the first few square ring and moderately reduced completely before reaching the design centre. In brief, the high-intensity EFD recorded a maximum limit value of 4000 V/m but MFD just reached a peak value of 150A/m. On the other hand, we came across a famous query that linked with the detection of EFD and MFD on the Rogers RO3006 material. This is because metal-composed material like MSM structure generally has a free electron in their physical structure. Even though any dielectric substrate has a free-electron deficiency, the EFD and MFD still occurred. This is because the metal structure and substrate material have delocalisation of electron oscillation which causes both phenomena to happen on the Rogers RO3006 material.

**Figure 6:**
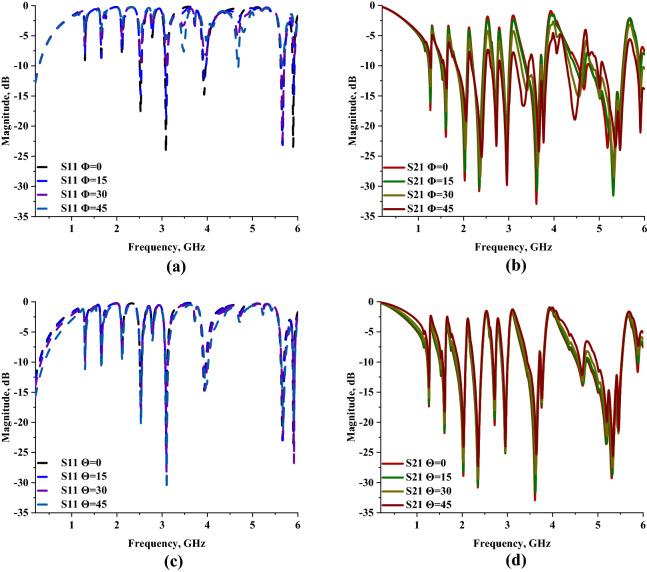
Phi and theta of 0° to 45°: **(a)** S11 Polarization, **(b)** S21 Polarization, **(c)** S11 Incident angle, **(d)** incident angle.

The effectiveness and compactness of the proposed MSM design can be computed by adopting the effective medium ratio (EMR) formula as expressed in Eq. (5). The equation is described as the ratio of wavelength and dimension of the metamaterial design. Meanwhile, the result should be more than 4 to meet the ideal value. Moreover, a metamaterial design that complies with this condition will obtain negative permittivity and/or permeability values. The MSM design possesses an optimised EMR value at 1.200 GHz, which was decreased for the following resonance frequencies. Table 4 demonstrates the EMR values for all resonance frequencies. Meanwhile, the polarization and incident angle results of the proposed metamaterial design were illustrated in Fig. 6a–d. Both analyses indicate that the variations in angle have a major influence on the outcomes and manifest dependent characteristics. The polarization-dependent values exhibit 25% discrepancies when compared to the proposed metamaterial S21 result. It also has similar discrepancies for the incident angle analysis at all four angles.

**Table 4:** EMR values of proposed MSM design at all resonance frequencies.

Frequency	1.200	1.458	1.560	1.896	2.268	2.683	2.940	3.580	5.872
EMR	25.000	20.576	19.231	15.823	13.228	11.182	10.204	8.380	5.109

5 $$EMR = Wavelength (\lambda )/Unit \, cell \, length \left(L\right)$$

Meanwhile, the SAR values averaged over 1 g and 10 g of tissue volumes, calculated using the CST software, with and without the metamaterial structure at all nine resonance frequencies which fall in the L-, S- and C-bands are tabulated in Tables 5 and 6. The human head phantom models in the tables graphically illustrate the maximum SAR values of each variable and frequency by using a yellow colour contour. Meanwhile, the least SAR value exposure is shown in the blue colour contour. Although the radiation exposure area is quite similar for both without and with metamaterial design, the level of exposure is greatly different from each other. For example, the exposure areas for 1 g of tissue volume at 1.458 GHz are relatively illustrating a similar amount, however, the maximum SAR values (in yellow contour) reduce respectively when using metamaterial design. From these tables, a clear observation can be made that most of the maximum SAR values occurred only on the cheek area of the right-hand side human head. Furthermore, we discovered that the SAR values averaged over 1 g and 10 g of tissue volume reduced in a similar pattern and has a reduction percentage mean value of 88.931% and 88.828%, respectively. The highest SAR value was recorded at the frequency of 1.560 GHz for both 1 g and 10 g, which was 98.140% and 98.283%, respectively. Besides that, the first seven resonance frequencies of the proposed metamaterial have successfully reduced the SAR values by more than 90%. Meanwhile, the last two resonance frequencies manifest SAR reduction values of more than 50%. In summary, the proposed MSM structure has complied with the objective of this research, which was to create a unique metamaterial design that shows overall SAR reduction values of more than 50%. Besides that, the proposed metamaterial is suitable for EM absorption reduction applications.

**Table 5:** SAR values of 1 g and 10 g of tissue volumes without and with the MSM design at L-band.

L-band resonance frequency	1 g		10 g	
	SAR value without metamaterial	SAR value with metamaterial	SAR value without metamaterial	SAR value with metamaterial
1.200				
	0.596	0.013	0.397	0.009
1.458				
	0.191	0.004	0.139	0.003
1.560				
	0.385	0.007	0.251	0.004
1.896				
	1.733	0.057	0.999	0.032

**Table 6:** SAR values of 1 g and 10 g of tissue volumes without and with the MSM design at S-band and C-band.

S- and C-band resonance frequency	1 g		10 g	
	SAR value without metamaterial	SAR value with metamaterial	SAR value without metamaterial	SAR value with metamaterial
2.268				
	1.508	0.088	0.813	0.046
2.683				
	1.208	0.080	0.579	0.038
2.940				
	0.592	0.033	0.283	0.018
3.580				
	0.654	0.160	0.283	0.070
5.872				
	1.867	0.884	0.666	0.321

## Unit cell and substrate material analysis

**Figure 7:**
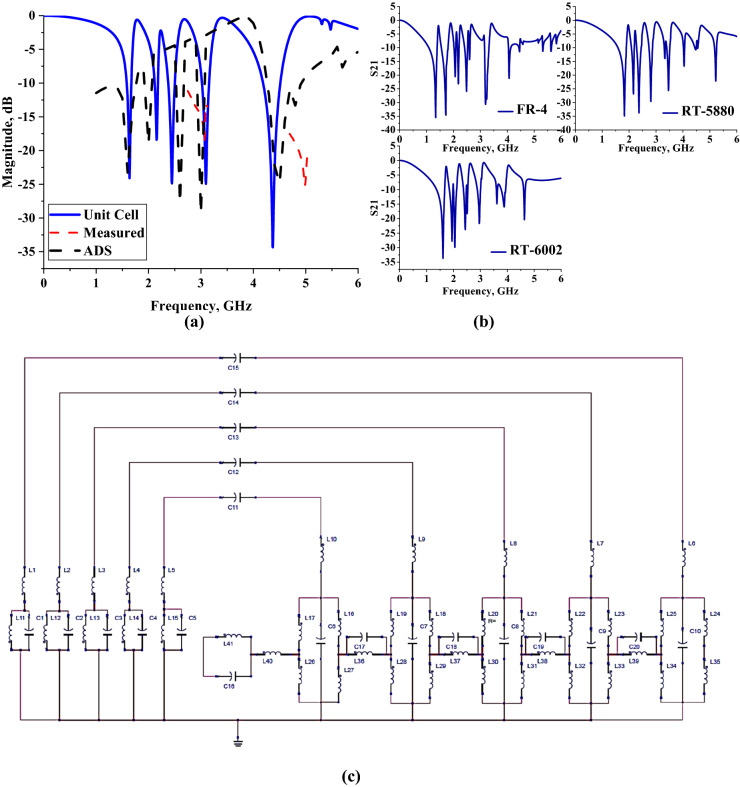
S21 results of: **(a)** Unit cell MSM design, **(b)** Several substrate materials analyses for proposed MSM design, (**c**) Equivalent circuit.

In this analysis, the unit cell result of the proposed metamaterial design was briefly discussed and the effects of various substrate material usage such as Rogers RT5880, FR-4, and Rogers RT6002 were examined for further clarifications. The unit cell design of the proposed metamaterial structure was simulated in CST software to compare with the S21 of the MSM design. Figure 7a illustrates the transmission coefficient result of the unit cell proposed MSM design. Meanwhile, Fig. 7b demonstrates the analysis of the few substrate materials that were utilized as a dielectric base material by adopting a similar MSM copper design structure. The unit cell MSM design exhibits only five resonance frequencies which start at 1.638 GHz. The lower band resonance frequencies are difficult to obtain in unit cell design as illustrated in Fig. 7a. The preliminary analysis of this research work indicates that the bigger substrate materials can manifest lower resonance bands for the square-shaped metamaterial design. However, the bigger metamaterial will face some difficulties for the design integration inside any miniaturized and compact wireless device. Meanwhile, the general array cell design approach for the EM absorption reduction also is limited by the size of the structure (number of columns and rows). Therefore, fulfilling the conflicting goals of lightweight design, lower bands with multi-frequency band operation, and high EM absorption values is a challenging task, requiring a unique and compact metamaterial design structure. The performance of double-layer metamaterial structure was first investigated to identify whether the stacking affects the resonance frequencies. The two-layered homogenous metamaterial structure exhibit nine resonance frequencies which are similar to the proposed MSM design, but has lower magnitude values at certain peak points. Due to the three-layered metamaterial design possessing multi-bands (L-, S-, and C-bands) with nine resonance frequencies and acceptable magnitude values, it was finally selected for the SAR reduction analysis.

On the other hand, experimental validations were analysed for the fourth and fifth resonance frequencies of the unit cell metamaterial design (demonstrated in Fig. 7a). The comparison of both simulation and measurement methods indicates a slight difference in the fourth resonance, while on the fifth peak point, the results majorly differ. The fabricated metamaterial manifest S21 results at 3.0667 and 4.986 GHz with magnitude values of − 18.433 and − 25.080 dB, respectively. The discrepancies between the two methods were occurred due to calibration error that has a great influence on the data produced. Besides that, the MSM design is small, unintentional flaws might have happened when removing excess copper from the substrate material. The simulation results of the unit cell metamaterial were further validated by the equivalent circuit model. The equivalent circuit model as illustrated in Fig. 7c possesses capacitive effects (C1 to C20) are denoted for the gaps between rings, while the inductive effect (L1 to L41) is for metallic strips. The constructed circuit model manifests resonance frequencies at 1.6, 2.0, 2.6, 3.0, and 4.5 GHz with magnitude values of − 23.3, − 18.9, − 27.7, − 29.3 and − 24.9 dB respectively. Meanwhile, the analysis usage of multiple substrate materials was conducted by utilising FR-4, Rogers RT-5880 and Rogers RT-6002 with an ε of 4.3, 2.2, 2.94 respectively. Few thicknesses of substrate material were adapted in this analysis, for example, 1.6 mm, 1.575 mm, and 1.524 mm, respectively for stated substrate material. Although these substrate materials have a bigger thickness, the metamaterial design structures exhibit either seven or eight resonance frequencies with almost tolerable magnitude values. In a nutshell, the proposed MSM design have better performance while possessing the least substrate thickness when combining all three layers by comparing it to these materials.

## SAR reduction analysis

Typically, the antenna inside a mobile phone will emit radiation and it will be absorbed by the tissue inside the human body. Therefore, to shield humans from most of the radiation, many methods were utilised in recent years. One of the most commonly used methods is the metamaterial structure where it will generally block or absorb the radiofrequency energy from entering the human body. Generally, the metamaterial structure has also been integrated with the mobile antenna to enhance the gain or bandwidth. However, the mobile phone that supports the fifth-generation network is composed of a compact high-performance antenna to satisfy the customer with the faster network coverage. Therefore, in this research work, the author focused only on the metamaterial structure that can be integrated inside the mobile phone without disturbing any existing parts. In this case, the metamaterial solely acts as a shield that protects humans from radiation. The amount of this energy that is absorbed by the human body tissues can be calculated using the SAR formula which is expressed as the power absorbed per unit mass. The SAR simulation in this parametric study utilised a similar standardized human head model filled with a specific liquid substance that is homogenous to an actual human head. In this section, using an imported cell phone model from CST, the simulation was done while operating at the highest power level for all the resonance frequencies. The parametric studies in this section were focused on different positions of metamaterial inside the mobile phone, the various distances between mobile phone to the voxel human head, and radiation reduction using unit cell metamaterial. Generally, the typical design structure identifies the type of metamaterial and what kind of electromagnetic properties are possessed. Therefore, the trial and error method was utilised in designing the metamaterial structure and finding the optimal position to reduce SAR value. Hence, the unique MSM design can reduce 98% of the SAR value emitted from the mobile phone.

### MSM design position inside the mobile phone

**Figure 8:**
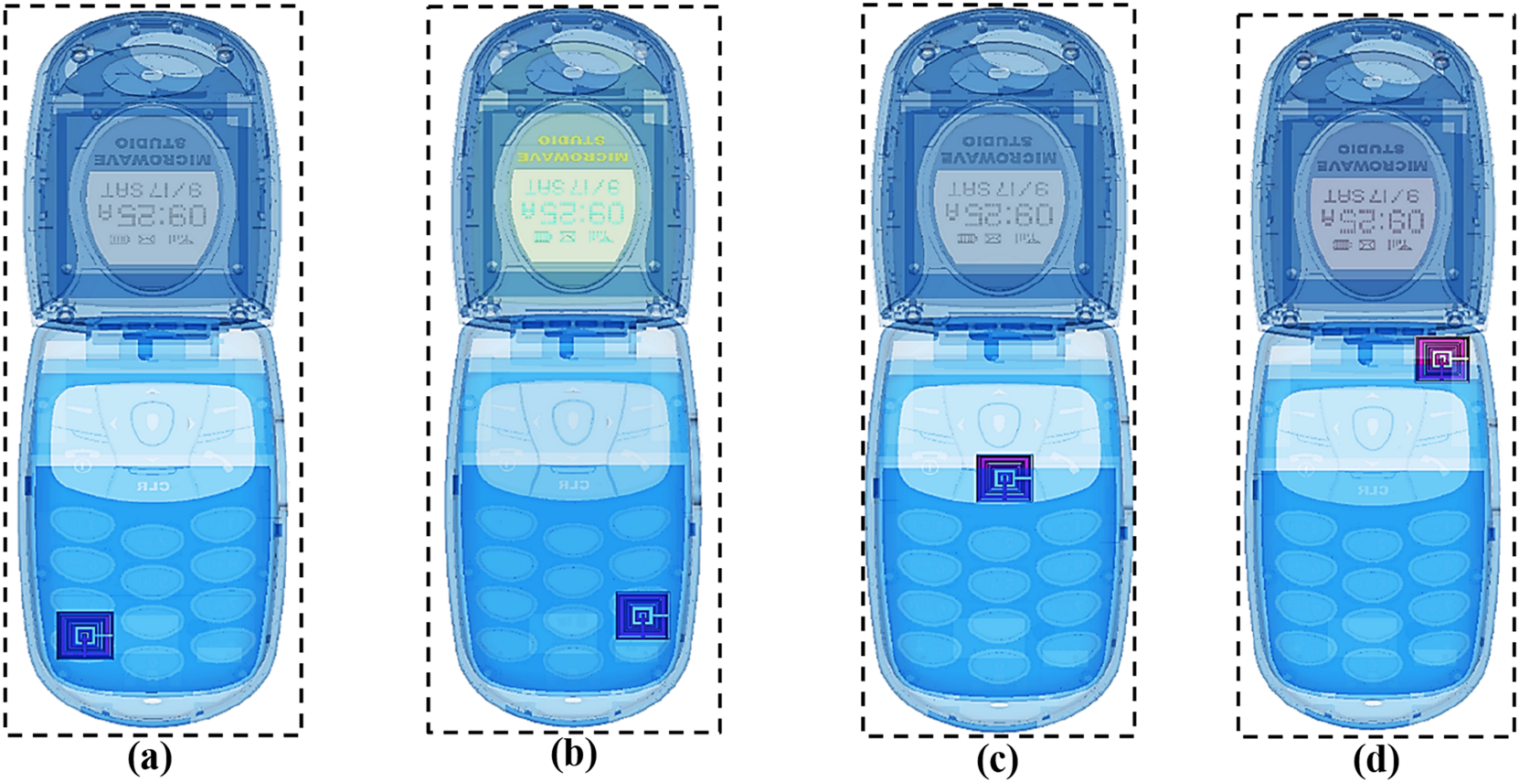
Multi-layer metamaterial structure in different positions inside the mobile phone: **(a)** Position 1, **(b)** Position 2, **(c)** Position 3, **(d)** Proposed.

Following the selection of metamaterial design, we investigated a few strategical positions of MSM structure inside the wireless device. This analysis was mainly focused on obtaining the most robust SAR reduction results by placing the proposed metamaterial design inside the mobile phone in different positions as demonstrated in Fig. 8a–d. MSM structure mostly was positioned all over the phone to find the variation in SAR reduction values. Tables 7 and 8 display the SAR values of four different positions for 1 g and 10 g of tissue volumes. For both tissue volumes, the metamaterial Positions 1 to 3 as in Fig. 8a–c exhibited similar patterns of SAR results. Meanwhile, Position 3 had the least SAR value for all the exhibited resonance frequencies. The MSM design is placed near to mobile antenna for the proposed position, and it indicates optimised results compare to the other three positions. This allows for the conclusion that the various SAR results were caused by the position of the metamaterial inside the mobile phone. However, the placements of metamaterial inside the phone are less clear-cut; hence, the trial-and-error method was also adopted in this simulation to achieve optimal results. Lastly, the position as shown in Fig. 8d was selected because it manifested optimised SAR values.

**Table 7:** SAR reduction values for 1 g of tissue volumes at various metamaterial positions.

SAR	1 g (W/kg)								
Frequency (GHz)	1.200	1.458	1.560	1.896	2.268	2.683	2.940	3.580	5.872
Position 1	0.600	0.197	0.389	1.733	1.579	1.227	0.534	0.680	1.809
Position 2	0.602	0.199	0.388	1.730	1.495	1.270	0.535	0.690	1.756
Position 3	0.599	0.198	0.386	1.737	1.551	1.151	0.611	0.657	1.842
Proposed	0.013	0.004	0.007	0.057	0.088	0.080	0.033	0.160	0.884

**Table 8:** SAR reduction values for 10 g of tissue volumes at various metamaterial positions.

SAR	10 g (W/kg)								
**Frequency (GHz)**	1.200	1.458	1.560	1.896	2.268	2.683	2.940	3.580	5.872
Position 1	0.402	0.138	0.253	1.000	0.848	0.586	0.264	0.296	0.643
Position 2	0.401	0.139	0.253	0.999	0.803	0.609	0.272	0.299	0.625
Position 3	0.399	0.136	0.251	1.003	0.834	0.550	0.300	0.284	0.655
Proposed	0.009	0.003	0.004	0.032	0.046	0.038	0.018	0.070	0.321

### Human head and mobile phone distances

In this parametric study, two different distances were analysed for the SAR reduction calculation with and without the MSM metamaterial design. The fundamental assumption of this study is that the longer the distance, the greater the calculated SAR values. Distances of 5 and 10 mm were examined, and the results are demonstrated in Tables 9 and 10. The comparisons between the SAR values with and without metamaterial reveal a few important details. The third resonance frequency that falls in the L-band recorded the highest SAR values for both 5 and 10 mm distance in 1 g and 10 g of tissue volumes. Meanwhile, the SAR values dropped gradually when the wireless device was shifted 5 mm away from the head. Subsequently, the values also decreased gradually when the distance was increased to 10 mm. Meanwhile, the least SAR value happened at the last resonance frequency of the C-band for both 5 and 10 mm distances in 1 g and 10 g of tissue volumes. At this frequency, the SAR reduction percentage value slightly increased in the 10 mm distance. Furthermore, the first resonance frequency at L-band has the second-highest SAR reduction values for 5 mm distance and increased slightly for the next limit. In addition to that, the first seven resonance frequencies have SAR reduction percentage values of more than 90% for 1 g and 10 g of tissue.

**Table 9:** 1 g SAR results with percentage values for various mobile distances from human head.

	SAR	1 g (W/kg)								
	Frequency (GHz)	1.200	1.458	1.560	1.896	2.268	2.683	2.940	3.580	5.872
5 mm	Without	0.419	0.108	0.220	1.011	0.956	0.664	0.322	0.397	1.289
	With	0.009	0.002	0.004	0.031	0.052	0.044	0.019	0.096	0.594
	Percentage	97.85	98.15	98.18	96.93	94.56	93.37	94.10	75.82	53.92
10 mm	Without	0.309	0.070	0.134	0.602	0.586	0.392	0.195	0.243	0.975
	With	0.006	0.002	0.002	0.017	0.031	0.026	0.011	0.059	0.447
	Percentage	98.06	97.14	98.51	97.18	94.71	93.37	94.36	75.72	54.15

**Table 10:** 10 g SAR results with percentage values for various mobile distances from human head.

	SAR	10 g (W/kg)								
	Frequency (GHz)	1.200	1.458	1.560	1.896	2.268	2.683	2.940	3.580	5.872
5 mm	Without	0.289	0.078	0.145	0.611	0.526	0.339	0.169	0.173	0.479
	With	0.006	0.002	0.002	0.018	0.028	0.022	0.010	0.042	0.224
	Percentage	97.92	97.44	98.62	97.06	94.68	93.51	94.08	75.72	53.24
10 mm	Without	0.216	0.050	0.090	0.368	0.334	0.202	0.104	0.112	0.380
	With	0.004	0.001	0.001	0.010	0.017	0.013	0.006	0.028	0.175
	Percentage	98.15	98.00	98.89	97.28	94.91	93.56	94.23	75.00	53.95

### SAR analysis with unit cell metamaterial

The unit cell metamaterial was simulated in CST software with a similar method as described in the computer simulation technique section. The unit cell metamaterial successfully manifests quintuple resonance frequencies, for instance, 1.638, 2.154, 2.448, 3.096, 4.374 GHz, respectively. The SAR percentage values for all resonance frequencies were tabulated in Table 11. The first four resonance frequencies also exhibit SAR values of more than 90%, while the last frequency has a higher than 50% value for both 1 g and 10 g of tissue volumes. Although the unit cell metamaterial as well manifests optimised SAR reduction values, the authors focus to produce resonance frequencies of more than five in the selected frequency range. Therefore, the multi-layered metamaterial structure was introduced in this research work.

**Table 11:** 1 g and 10 g SAR results with percentage values for unit cell metamaterial.

SAR	1 g (W/kg)					10 g (W/kg)				
Frequency (GHz)	1.638	2.154	2.448	3.096	4.374	1.638	2.154	2.448	3.096	4.374
WTM	0.684	1.074	1.456	0.304	0.345	0.436	0.574	0.730	0.108	0.125
WM	0.012	0.053	0.089	0.023	0.160	0.007	0.027	0.043	0.008	0.058
%	98.25	95.07	93.89	92.43	53.62	98.39	95.30	94.11	92.59	53.60

*WTM* without metamaterial, *WM* with metamaterial.

## Comparison of previously reported SAR reduction studies

Table 12 illustrates the existing SAR reduction comparison research works as well as the introduced MSM structure. The entire references stated here are successfully designed metamaterial structures exclusively for either SAR reduction or absorption applications. Moreover, all earlier research studies examined here also possess identical double-negative behaviour except proposed metamaterial. However, the superior electromagnetic property of all time, known as the left-handed characteristic, was successfully claimed in this research work. Besides unique electromagnetic properties, unconventional material provides additional benefits likely, good elasticity, strength, or heat conduction. Approximately all the previous works used copper material to construct the metamaterial structure because this economical material possesses superior conductivity besides gold and silver. Moreover, along its way, the copper material transmits signals without losing electricity. Even though several works indicate enhanced SAR reduction values, they show several setbacks. The works disclose that the left-handed metamaterial is generally hard to gain for all kinds of metamaterial designs. Meanwhile, stated references clearly show that the proposed split ring resonator design exhibits only negative permittivity and permeability values. It has something to do with the fact that, the configurations of the split between rings and a distinct number of rings have a significant impact on the results. Besides that, the square-shaped metamaterial structure manifests lower resonance frequencies with greater current distribution. Overall, the structure also has a practicable natural frequency that can be tuned by modifying the configuration of the rings. Meanwhile, compact-sized electronic devices have turned into the latest development in the scientific field. The references^31–34^ have larger array cells that become a primary restriction for the application of SAR reduction analysis. Due to this limitation, we handle the metamaterial size miniaturization by providing reasonable interest when designing a unit cell metamaterial structure. In the meantime, metamaterial design is also applied for absorption applications like in the reference^35^. Although the author of this reference, has successfully produced a high absorber metamaterial design, it also suffers from size miniaturization limitations. Meanwhile, recently published absorption related research works^36–38^ also mostly applied for frequency range more than 6 GHz. If the works cover the sub-6 range than, the authors proposed complex or bigger substrate material. Furthermore, there are only a few analyses regarding 5G Mobile Network radiation reduction have been investigated in recent years. The metamaterial structures of the stated references in Table 12 owned either single or double resonance frequencies as in^31–33^. In these studies, the metamaterial design has satisfactory absorption reduction values, but we aimed to obtain resonance frequencies more than dual-band. Therefore, the compact stack layered square-shaped metamaterial structure was introduced for SAR reduction application, precisely attributed to the fifth-generation technology. The homogenous thin multi-layered metamaterial design for SAR reduction in mobile phones becomes the novelty of this paper.

**Table 12:** A comparison between proposed metamaterial design and previous studies.

References	Resonance frequencies (GHz)	Band	Application	Size (mm)	Characteristic
^31^	0.9 and 1.8	Double	SR; 1 g: 43% and 44%	AC: 44.4 × 33.3	Double-negative
^32^	2.1	Single	SR; 1 g: 35.93%	AC: 187 × 117	Double-negative
^35^	13.58, 14.82, 16.65 and 18.44	Multi	A; 79%, 99%, 88%, and 92%	AC: 220 × 220	Double-negative
^33^	0.9 and 1.8	Double	SR; 1 g: 40% and 49%	AC: 54.72 × 39	Double-negative
^34^	0.9 and 1.8	Double	SR; 1 g: 14.23% and 15.30%	AC: 36 × 36	Double-negative
Proposed	1.200, 1.458, 1.56, 1.896, 2.268, 2.683 2.940, 3.580 and 5.872	Triple	SR; 1 g: 97.82%, 97.71%, 98.14%, 96.72%, 94.18%, 93.34%, 94.34%, 75.49%, and 52.64%	MSM: 10 × 10	Left-handed

*SR* SAR reduction, *A* absorption, *AC* array cell.

## Conclusion

This paper concludes by arguing the SAR values computed using CST software with the attachment of MSM design in the mobile phone. Concern about RF exposure to humans increases tremendously because of the fast growth in the usage of mobile phones. Hence, a reduction of radiation exposure from a mobile phone is highly recommended in the current research field. Specific Anthropomorphic Mannequin phantom was used in this simulation to compute the SAR values numerically. The outcomes of the proposed MSM structure reveals acceptable magnitude values with multi-bands nonuple resonance frequencies. Each band, namely L-, S-, and C-bands have 4:4:1 separated resonance frequencies precisely at 1.200, 1.458, 1.560, 1.896, 2.268, 2.683 2.940, 3.580 and 5.872 GHz, respectively. The possibility to neglect the discrepancy between both simulated results was very high because the difference is less than 5 GHz. Furthermore, the lowest SAR value reported was higher than 50% at 5.872 GHz for both average over 1 g and 10 g of tissue volumes. The exhibited frequency bands can be used in a wide area of applications, for example, wireless headphones (Bluetooth), garage door openers, keyless vehicle locks, etc. Finally, the obtained data accomplished the aim of this investigation where the SAR values of the proposed metamaterial structure reduced extraordinarily and have superior characteristics.
